# Matters arising: expanding the literature base on blunt trauma-induced abdominal wall hernia

**DOI:** 10.1186/s12245-026-01387-8

**Published:** 2026-09-16

**Authors:** Mohamed Hajri, Lassad Gharbi, Zied Hadrich

**Affiliations:** 1 Faculty of Medicine of Tunis, Department of General Surgery, Mongi Slim University Hospital,University of Tunis El Manar, Sidi Daoud, Tunis, 1006 Tunisia; 2 Laboratory of Genetics, Immunology, and Human Pathologies LR05ES05, Faculty of Sciences of Tunis, University of Tunis El Manar, Tunis, Tunisia

To the Editor,

We read with great interest the recent article by Ben Ismail et al., entitled “Blunt trauma-induced abdominal wall hernia with small bowel incarceration: case report and review of the literature,” published in the *International Journal of Emergency Medicine* [1]. The authors should be commended for reporting a rare and clinically important presentation of traumatic abdominal wall hernia (TAWH), complicated by small bowel incarceration, and for emphasizing the need for prompt computed tomography and timely surgical management.

We would like, however, to draw attention to additional previously published cases that were not mentioned in the narrative review.

In 2016, our department reported a case of post-traumatic anterior abdominal wall hernia in the *Pan African Medical Journal* [2]. Although this case was not complicated by bowel obstruction or strangulation, it remains directly relevant to the broader spectrum of TAWH following blunt trauma. The patient was a 32-year-old man with a body mass index of 30 kg/m² who was involved in a road traffic accident, with abdominal crushing against the steering wheel. Despite the severity of the mechanism, the abdominal wall hernia was not detected on initial physical examination, probably because the emergency assessment was focused on intra-abdominal injury and because the patient’s elevated BMI made the clinical examination less informative. Computed tomography revealed an 8-cm anterior abdominal wall defect with bowel loops located immediately beneath the skin. Operative exploration found a 12-cm musculoaponeurotic defect, with small bowel loops covered only by parietal peritoneum, as well as an associated mesenteric injury. The abdominal wall defect was repaired by interrupted sutures. The postoperative course was complicated by infected skin necrosis, which evolved favorably with directed wound care [2].

This observation illustrates several points that complement the discussion by Ben Ismail et al. First, TAWH may be clinically occult, even when the defect is large, and therefore requires a high index of suspicion after blunt abdominal trauma. This is particularly relevant in overweight or obese patients, in whom inspection and palpation of the abdominal wall may underestimate the extent of musculoaponeurotic disruption. Therefore, after a violent blunt abdominal trauma, especially involving deceleration, crushing, or direct impact against the steering wheel, CT imaging should play a central role even when the clinical examination is poor or non-specific. Second, computed tomography is not only useful for diagnosis, but also essential for identifying associated intra-abdominal injuries and defining the anatomy of the abdominal wall defect. Third, the decision between immediate and delayed repair should be individualized according to the patient’s hemodynamic status, associated injuries, contamination risk, and the presence or absence of bowel incarceration or strangulation. Finally, anterior abdominal wall defects caused by deceleration or compression mechanisms may represent a distinct subgroup of TAWH, with specific diagnostic and therapeutic considerations.

Two additional reports cited in our 2016 article also deserve mention in the context of a literature review on TAWH. Hassen et al. described a handlebar hernia, highlighting a localized blunt-force mechanism responsible for abdominal wall disruption [3]. Drago et al. reported a traumatic ventral hernia and discussed surgical treatment strategies [4]. While these reports differ from the case presented by Ben Ismail et al. in terms of mechanism, location, and clinical severity, they remain part of the cumulative literature on post-traumatic abdominal wall defects and may contribute to a more comprehensive overview of diagnostic pitfalls and surgical options.

We acknowledge that the review by Ben Ismail et al. focused particularly on TAWH complicated by bowel obstruction, as reflected by the cases summarized in their table. For this reason, the 2016 PAMJ case and the two additional reports mentioned above may not necessarily belong in a table restricted to obstructive presentations. Nevertheless, the article also discusses the broader epidemiology, classification, diagnosis, and management of TAWH. Within that broader narrative framework, these earlier cases are relevant because they reinforce the rarity, heterogeneity, and potential under-recognition of TAWH in the emergency setting.

The absence of these cases may have implications beyond bibliography alone. It may influence how readers perceive the available evidence, particularly in a field where knowledge is largely based on case reports and small series. Including additional reports from different clinical settings may help refine the understanding of TAWH mechanisms, radiological findings, operative indications, and postoperative morbidity. This is especially important because standardized diagnostic and therapeutic algorithms remain limited, and each well-documented case can contribute meaningfully to clinical decision-making.

In conclusion, we congratulate Ben Ismail et al. for their valuable contribution to the literature on traumatic abdominal wall hernia. We suggest that the previously reported case from our department, together with the earlier cases cited therein, should be considered when discussing the broader literature on TAWH. Their inclusion would strengthen the comprehensiveness of the review and further support the authors’ central message: TAWH should be suspected after blunt abdominal trauma, even when clinical findings are subtle, and computed tomography plays a pivotal role in diagnosis and management planning.

## Data Availability

No datasets were generated or analysed during the current study.

## Abbreviations

BMI: Body mass index
CT: Computed tomography
TAWH: Traumatic abdominal wall hernia

## References

[1] Ben Ismail I, Sghaier M, Zenaidi H, Messoudi H, Rebii S, Zoghlami A. Blunt trauma-induced abdominal wall hernia with small bowel incarceration: case report and review of the literature. Int J Emerg Med. 2026;19:34. 10.1186/s12245-026-01127-y. 41612183 PMC12853694

[2] Mzoughi Z, Bayar R, Khmiri H, Gharbi L, Khalfallah MT. Hernie post traumatique de la paroi abdominale antérieure. Pan Afr Med J. 2016;24:203. 10.11604/pamj.2016.24.203.9517. 27795798 PMC5072850

[3] Hassan KAF, Elsharawy MA, Moghazy K, AlQurain A. Handlebar hernia: a rare type of abdominal wall hernia. Saudi J Gastroenterol. 2008;14(1):33–5. 19568493 10.4103/1319-3767.37805 PMC2702886

[4] Drago SP, Nuzzo M, Grassi GB. Traumatic ventral hernia: report of a case, with special reference to surgical treatment. Surg Today. 1999;29(10):1111–4. 10.1007/s005950050655. 10554341

